# Clinical characteristics of patients with diabetic polyneuropathy: the role of clinical and electromyographic evaluation and the effect of the various types on the quality of life

**DOI:** 10.1111/j.1742-1241.2008.01730.x

**Published:** 2008-07

**Authors:** N Ovayolu, E Akarsu, E Madenci, S Torun, O Ucan, M Yilmaz

**Affiliations:** 1 Gaziantep University Health Sciences SchoolGaziantep, Turkey; 2 School of Medicine, Departments of Endocrinology and MetabolismGaziantep, Turkey; 3 Department of Physical TherapyGaziantep, Turkey; 4 Department of Neurology, Sahinbey Medical CenterGaziantep, Turkey

## Abstract

**Objective:**

This study was performed to identify the relationship between the quality of life and polyneuropathy which is one of the complications of diabetes.

**Methods:**

Total 111 patients with diabetes mellitus were taken into the study as type 1 and type 2. Patients were accepted having polyneuropathy according to their electroneuromyography (ENMG) results. To evaluate the quality of life in the patients Short Form 36 (SF-36) and World Health Organization Quality of Life Questionnaire abbreviated version (WHOQOL-BREF) were used.

**Results:**

Clinical polyneuropathy was found in 46% of the patients, while polineuropathy was found in 63% of the patients with evaluation ENMG. The patients with polyneuropathy had poor quality of life according to SF-36 and WHOQOL-BREF (p < 0.001). The mean quality of life scores of patients who had sensoriomotor and mix polyneuropathy, were lower than sensory type and axonal polyneuropathy.

**Conclusion:**

Diabetic polyneuropathy influences the quality of life in a negative way. The quality of life scores of patients who had polyneuropathy continuing with mixed pathogenesis and sensoriomotor type, become worse for this reason, even if the patients do not have any clinical polyneuropathy, this being evaluated with ENMG.

**What's known**

It is now widely accepted that the goals of therapy in patients with chronic disease are not only to improve survival, but also to improve quality of life. Diabetic neuropathy negatively affects the quality of life of the patients.

**What's new**

Evaluation of ENMG is important for the early diagnosis of neuropathy. Taking necessary precautions according to the results of ENMG will be useful for improving the quality of life.

## Introduction

Diabetes mellitus is associated with high-risk complications, essentially micro- and macrovascular complications (1). The neuropathies are among the most common of the long-term complications of diabetes, affecting up to 50% of patients (2). The most common type of diabetic neuropathy is a mixed (both motor and sensory), symmetrical, distal and primarily sensory polyneuropathy (3).

Electrodiagnostic testing plays a key role in the characterization of neuropathies (4) and its use in the diagnosis of diabetic polyneuropathy represents an extension of the clinical examination and include studies of sensory and motor nerve conduction, late response recordings such as of the F wave, and needle electromyography. Electrodiagnostic findings should confirm the clinical findings and in some cases allow the detection of subclinical abnormalities (3).

Diabetic neuropathies cause morbidity with significant impact on the quality of life patients with diabetes (5–7). The quality of life is one of the most important indications, being very important for health, and it is a measure of physical–social functions and physical and spiritual wellbeing. Patients with diabetes lead low-quality lives when compared with healthy individuals. It was reported that the most important factors being effective on the quality of life in these patients are the complications progressing during the course of the illness was identified (8). It is now widely accepted that the goals of therapy in patients with chronic disease are not only to improve survival, but also to improve quality of life. Investigation of patient quality of life may provide useful information to set up standards for the process of medical and nursing care (9). Advanced practice nurses are in a unique position to implement strategies for the prevention of serious and debilitating complications from diabetic neuropathy, including foot assessment, education and specialist referrals (10). Our study was conducted to evaluate the effect of diabetic polyneuropathy, being one of these complications, on the quality of life.

## Methods

This investigation was carried out between 2004 and 2005 on diabetic patient who applied to the department of endocrinology.

### Study sample

At the first stage of the investigation, consent was provided by patients who will participate in the study. Patients who had microalbuminuria were included in the study (albumin excretion rate of 20–200 μg/min (30–300 mg/day). Microalbuminuria is an important clinical finding because it is not only associated with an increased risk of progression to overt proteinuria (macroalbuminuria) and renal failure, but also cardiovascular events. In patients who progress to overt nephropathy, microalbuminuria usually precedes macroalbuminuria by an interval of 5–10 years (11). The presence of diabetic polyneuropathy in the patients was evaluated both clinically and with electroneuromyography (ENMG). Patients were accepted having polyneuropathy according to their ENMG results.

### The form of questionnaire

The questionnaire form containing information related to data about sociodemographical features were formed by the researchers for data collection presenting the existence of microalbuminuria, and the level of HbA1c. A Short Form 36 (SF-36) and World Health Organization Quality of Life Questionnaire abbreviated version (WHOQOL-BREF) consisting of 26 questions were used.

The Medical Outcomes SF-36 was used to evaluate quality of life. The SF-36, which was developed by Ware and Sherbourne (12), assesses eight health concepts: physical functioning (PF), role limitations because of physical problems (RP), social functioning (SF), role limitations due to emotional problems (RE), mental health (MH), vitality (VT), bodily pain (BP) and general health (GH) perception. In this study, the quality of life was evaluated as three mean dimensions like GH perception, functional status (PF + RP + SF + RE), wellness (MH + VT + BP) and global score. Thirty-six substances take place in the measure while two substances of the questions were being answered in the form of yes/no, and the others are in the form of ‘Likert’ (with three or with six). All raw scale scores were linearly converted from zero (worst possible health status or quality of life) to 100 (best possible health status or quality of life). The score of the subgroups as well as the final global score of the SF-36 changes between 0 and 100, respectively and higher score mean good quality of life. These measures provide a speed evaluation and it was filled in 5–10 min (12–14). Different language versions of the SF-36 are available, including Turkish. Pinar (13) performed SF-36’s validation in Turkish patients (1995). In her study the test–retest correlation was 0.94 and Cronbach alpha value was 0.92.

As there were no questions about sexual function in SF-36, it was used together with WHOQOL-BREF, which also measures quality of life (15,16). WHOQOL-BREF which was composed of four domain factors (physical, psychological, social relations and the environment), was used to assess quality of life. Each of four domains had a possible score ranged between zero (poor quality of life) and 20 (excellent quality of life) and higher score mean good quality of life. WHOQOL-BREF was developed by WHO as a measure for the life of quality. Twenty-six questions being in the type of Likert take a place in the measure (17). The study of validity and reliability was performed for the Turkish population in 1999 by Fidaner et al. (18). The values of Cronbach alpha which is calculated in the study of reliability range between 0.53 and 0.83.

### Evaluation of diabetic polyneuropathy

#### Clinical evaluation

In the neurological evaluation performed by neurologist, with hypoesthesia of gloves-socks, hyporeflex or areflex of biceps, pathella or achilles and motor loss at least in two extremities were accepted as having clinically polyneuropathy.

#### Electroneuromyographical evaluation

Study of ENMG was performed by the Keypoint DANTEC device (Skovlunde, Denmark). Stimulation duration was 0.2 ms for motor, and 0.1 ms for sensory stimuli. All stimulations were performed supramaximally. Bipolar stimulus electrodes were used for all stimuli. Sensory examinations were all performed using the antiradical method. The band of frequencies was 20 Hz to 10 kHz in the sensory, motor and F-wave examinations. Nerve conduction velocity (NCV) under limit for the upper extremity was accepted as 50 m/s for motor conduction velocity (MCV) and 43 m/s for sensory conduction velocity (SCV). Also, NCV under limit for the lower extremity was 42 m/s MCV and SCV. Under limit the amplitude of motor unit potential (MUP) was taken as 6 mV for the median and ulnar nerve, 3 mV for peroneal nerve and 4 mV for tibial nerve. The amplitude of the sensory nerve action potential was accepted as 10 μV for the median and ulnar nerves and as 6 μV for the sural and peroneal superficial nerves. The tibial F-wave upper limit was accepted as 55 ms. Polyneuropathy was divided into three groups as motor, sensory and sensoriomotor electro physiologically according to involvement of the sensory and motor nerves. The median nerve motor distal latency over 4.4 ms and the SCV lower than 42 m/s and fourth finger median-ulnar sensory peak difference > 0.5 m/s were accepted as electrophysiological carpal tunnel syndrome (CTS). In respect of the pathogenesis of polyneuropathy the phenomena was divided into three groups according to the following criteria; these were demyelization polyneuropathy, axonal polyneuropathy or mixed polyneuropathy. Prolongation of motor distal latency more than 30% of normal, decrease of conduction velocity more than 25% of normal, prolongation of F wave more than 55 ms conduction block (so that the rate of proximal/distal amplitude MUP is under 50%) or temporal dispersion (so that the rate of proximal/distal MUP duration is more than 1.15) were evaluated as demyelization polyneuropathy. Decrease of motor and sensorial amplitude more than 40% of normal value and/or presence denervation potentials (fibrillation potential and positive sharp waves) in the needle ENMG were evaluated as axonal polyneuropathy (19).

### Statistical evaluation

The statistical package for social science (spss; SPSS, Chicago, IL, USA) for windows was used for statistical analysis. For comparison between distribution of sociodemographic features and incidence of polyneuropathy chi-squared test, quality of life and incidence of polyneuropathy Student *t-*test, quality of life and type of polyneuropathy, classification of polyneuropathy and presence of microalbuminuria Mann–Whitney *U*-tests were used. Linear regression analysis was used to investigate the most significant factor for poor quality of life. The parametric test was selected as the tests indicating normal distribution, and non-parametric tests were selected as those which did not indicate normal distribution. The level of significance was set at p < 0.05.

## Results

A total of 111 patients (66 females and 45 males) were included in the study; 21 of them were with type 1 diabetes and 90 with type 2 diabetes. The mean age of patients was 53.1 ± 12.3 years. The sociodemographical features of diabetic patients are given in the Table 1. Polyneuropathy was found in the 63% of the patients, but was not found in 37% of the patients. In the group with polyneuropathy, clinical polyneuropathy and duration of diabetes were found to be more significant (p < 0.05). No significant difference was found between age, gender, diabetes types, HbA1c levels and microalbuminuria with polyneuropathy (p > 0.05). But the frequency presence of polyneuropathy was found to be higher in female patients. Sensoriomotor type of polyneuropathy was found in 82% of the patients, while 19% of them had sensory type of polyneuropathy. However, there were no patients with motor type polyneuropathy. In respect of the pathogenesis of polyneuropathy, 73% of patients had axonal polyneuropathy, while 27% had mixed type polyneuropathy. There were no patients with demyelination polyneuropathy.

**Table 1:** Distribution of sociodemographic features according to the incidence of polyneuropathy

	Polyneuropathy			
Features	Positive (%)	Negative (%)	Total (%)	p
**Age (years)**				
18–35	5 (9.8)	5 (8.3)	10 (9)	0.897
36–53	20 (39.2)	26 (43.3)	46 (41.5)	
54 and ↑	26 (51)	29 (48.4)	55 (49.5)	
**Gender**				
Female	48 (69)	18 (44)	66 (60)	0.199
Male	22 (31)	23 (56)	45 (40)	
**Duration of diabetes**				
10 years ↓	16 (31)	38 (63)	54 (49)	0.001
10 years ↑	35 (69)	22 (37)	57 (51)	
**Type of diabetes**				
Type 1	10 (14)	11 (27)	21 (19)	0.085
Type 2	60 (86)	30 (73)	90 (81)	
**Clinical polyneuropathy**				
Positive	51 (73)	–	51 (46)	0.001
Negative	19 (27)	41 (100)	60 (54)	
**HbA1c**				
< 7	30 (43)	12 (30)	42 (38)	0.110
≥ 7	40 (57)	29 (70)	69 (62)	
**Microalbuminuria**				
Positive	14 (20)	4 (10)	18 (16)	0.125
Negative	56 (80)	37 (90)	93 (84)	
Total (%)	70 (63)	41 (37)	111 (100)	

The relationship between the mean effect of the quality of life of polyneuropathy are given in the Table 2. Mean effects of the quality of life of patients with the polyneuropathy was significantly lower than that of patients without polyneuropathy (p < 0.001). The subparameters of SF-36 and WHOQOL-BREF quality of life measures such as functional, wellbeing, general, global, physical, psychologic, social and environmental were found to be low in the polyneuropathy group (p < 0.001).

**Table 2:** Comparison of the SF-36 and WHOQOL-BREF quality of life measurements according to incidence of polyneuropathy

	Polyneuropathy		
The mean quality of life	Present (*n* = 70)	Absent (*n* = 41)	p
**SF-36**			
Functional	34.54 ± 2.85	62.76 ± 4.62	0.001
Wellbeing	32.92 ± 2.21	56.38 ± 3.16	0.001
General	34.41 ± 2.20	50.87 ± 3.13	0.001
Global	33.72 ± 1.88	57.34 ± 2.93	0.001
**WHOQOL-BREF**			
Physical	9.63 ± 0.37	13.95 ± 0.44	0.001
Psychological	12.02 ± 0.36	14.04 ± 0.42	0.001
Social	11.39 ± 0.34	14.30 ± 0.51	0.001
Environmental	11.83 ± 0.31	14.06 ± 0.39	0.001

SF-36, Short Form 36; WHOQOL-BREF, World Health Organization Quality of Life Questionnaire abbreviated version.

The quality of life parameters between sensorial and sensoriomotor polyneuropathy groups are compared in Table 3. No significant difference was found between them (p > 0.05).

**Table 3:** Comparison of the patients’ mean quality of life according to the type of polyneuropathy

	Polyneuropathy type		
The mean quality of life	Sensoriomotor (*n* = 57)	Sensorial (*n* = 13)	p
**SF-36**			
Functional	33.3 ± 3.0	40.5 ± 7.7	0.502
Wellbeing	32.4 ± 2.3	39.8 ± 5.6	0.261
General	34.3 ± 2.6	38.9 ± 4.3	0.326
Global	33.3 ± 2.1	38.3 ± 4.0	0.287
**WHOQOL-BREF**			
Physical	9.9 ± 0.4	9.0 ± 0.8	0.486
Psychological	11.7 ± 0.3	13.1 ± 0.7	0.063
Social	11.4 ± 0.3	11.3 ± 0.7	0.915
Environmental	11.6 ± 0.3	12.1 ± 0.5	0.544

SF-36, Short Form 36; WHOQOL-BREF, World Health Organization Quality of Life Questionnaire abbreviated version.

Comparison of the axonal and mix-type polyneuropathy groups is also made in Table 4, and no significant difference was found between the two groups (p > 0.05). No significance was found in the quality of life parameter, functional subunits of SF-36 and in the environmental subparameters of WHOQOL-BREF, in respect of the presence of microalbuminuria (p > 0.05). Wellbeing, general and global parameters of SF-36, and the physical, psychologic and social parameters of WHOQOL-BREF were found to be significant (p < 0.05). To find out the basic factors which reduce the quality of life, triple cross tables were constructed because of the low quality of life of patients with polyneuropathy and microalbuminuria. Statistical analysis demonstrated that the most basic factor affecting the quality of life of patients with microalbuminuria was polyneuropathy (Table 5).

**Table 4:** Comparison of the patients’ mean quality of life according to the classification of polyneuropathy

	Classification of polyneuropathy		
The mean quality of life	Axonal (*n* = 51)	Mix (*n* = 13)	p
**SF-36**			
Functional	36.1 ± 3.4	30.5 ± 4.9	0.402
Wellbeing	35.2 ± 2.6	30.0 ± 4.0	0.345
General	34.2 ± 2.4	32.8 ± 5.6	0.701
Global	34.8 ± 2.2	32.8 ± 4.0	0.602
**WHOQOL-BREF**			
Physical	9.7 ± 0.4	9.5 ± 0.7	0.995
Psychological	12.3 ± 0.3	11.1 ± 0.6	0.118
Social	11.7 ± 0.4	10.6 ± 0.5	0.170
Environmental	12.0 ± 0.3	10.8 ± 0.5	0.065

SF-36, Short Form 36; WHOQOL-BREF, World Health Organization Quality of Life Questionnaire abbreviated version.

**Table 5:** Comparison of the quality of life measurements of patients according to the presence of microalbuminuria

	Microalbuminuria		
The mean quality of life	Present (*n* = 18)	Absent (*n* = 93)	p
**SF-36**			
Functional	38.1 ± 7.74	46.2 ± 2.9	0.133
Wellbeing	30.0 ± 5.0	44.7 ± 2.3	0.014
General	32.3 ± 5.8	42.0 ± 2.0	0.031
Global	33.5 ± 5.04	44.1 ± 2.0	0.035
**WHOQOL-BREF**			
Physical	8.6 ± 8.8	11.7 ± 0.3	0.001
Psychological	11.3 ± 0.8	13.1 ± 0.3	0.027
Social	10.0 ± 0.8	12.9 ± 0.3	0.002
Environmental	11.7 ± 0.6	12.8 ± 0.2	0.322

SF-36, Short Form 36; WHOQOL-BREF, World Health Organization Quality of Life Questionnaire abbreviated version.

According to the results of linear regression analysis; the WHOQOL-BREF scores on each of the domains and SF-36 global score were low in polyneuropathy (p < 0.05). SF-36 global score and physical parameter of WHOQOL-BREF were low on the patients who had diabetes for more 10 years, social and physical parameter of WHOQOL-BREF were low in presence of microalbuminuria, SF-36 global and psychologic domain score of WHOQOL-BREF were low in female (p < 0.05) (Table 6).

**Table 6:** Results of linear regression analysis*

	Unstandardized coefficients		Standardized coefficients		
Parameters	*B*	SE	β	*t*	p
**SF-36**					
*Global*					
Constant	16.52	8.25	–	2.003	0.048
Polyneuropathy†	19.38	3.36	0.461	5.767	0.000
Duration of diabetes	−4.96	1.89	−0.210	−2.627	0.010
Female gender	6.96	3.26	0.168	2.133	0.035
**WHOQOL-BREF**					
*Physical*					
Constant	10.51	1.52	–	6.89	0.000
Polyneuropathy	3.69	0.58	0.48	6.30	0.000
Microalbuminuria	−2.36	0.74	−0.23	−3.19	0.002
Duration of diabetes	−0.76	0.32	−0.17	−2.32	0.022
*Psychological*					
Constant	9.44	0.87	–	10.84	0.000
Polyneuropathy	0.63	0.20	0.28	3.12	0.002
Female gender	1.33	0.56	0.21	2.35	0.020
*Social*					
Constant	11.70	1.29	–	9.04	0.000
Polyneuropathy	2.66	0.58	0.38	4.59	0.000
Microalbuminuria	−2.47	0.75	−0.27	−3.26	0.001
*Environmental*					
Constant	9.60	0.74	–	12.86	0.000
Polyneuropathy	2.22	0.51	0.38	4.32	0.000

* Several factors (age, female gender, duration of diabetes, type of diabetes, clinical polyneuropathy, HbA1c, microalbuminuria, polyneuropathy, type of polyneuropathy and classification of polyneuropathy) were tested for regression analysis. Only significant parameters are given.

† Polyneuropathy: according to ENMG. SF-36, Short Form 36; WHOQOL-BREF, World Health Organization Quality of Life Questionnaire abbreviated version.

## Discussion

Diabetic neuropathy is one of the most frequently encountered complications of diabetes mellitus (6,7). Previous studies indicated that diabetic neuropathy progressed in the diabetic patients ranging from 16% to 50% (20–24). In our study, according to neurological evaluation performed by neurologist clinical polyneuropathy was found in 46% of the patients, while polineuropathy was found in 63% of the patients with evaluation ENMG.

Some studies indicated that advanced age and the long duration of diabetes increased the prevalence of polyneuropathy (25–28); however, no relationship between the diabetic polyneuropathy and gender could be found (25,26). Daousi et al. (29) could not find any relationship between age, type of diabetes, gender, duration of the illness and the frequency of peripheral neuropathy with chronic pain. According to the results of our study, no significant relationship was found between the frequency of polyneuropathy and gender and type of diabetes. However, the frequency of presence of polyneuropathy was found to be higher in female patients and patients having diabetes for more than 10 years. The cause of polyneuropathy frequently seen in female patients can be explained by the fact that female patients have longer duration of diabetes than their male counterparts.

Electrophysiological evaluation is the most objective method defining subclinical and clinical neuropathy (30). In our study, although patients did not have clinical polyneuropathy, the presence of polyneuropathy in ENMG indicated the importance of ENMG in the early diagnosis of polyneuropathy.

Sensoriomotor polyneuropathy was demonstrated 82% of the patients in our study, whereas 19% of patients had the sensory polyneuropathy. Lloyd et al. (31) demonstrated that 12% of the patients with the type 2 diabetes had peripheral sensory neuropathy. Kastenbaur et al. (23) detected sensoriomotor neuropathy in 48% of diabetic patients with abnormal ankle reflex. The primary problem in the diabetic polyneuropathies was known to be mostly axonal polyneuropathy (32). In our study, the percentage of axonal polyneuropathy phenomena complies with the literature.

Several studies have demonstrated that diabetes negatively influenced the quality of life (33,34) and that polyneuropathy progressing because of diabetes worsens the quality of life (6,7,35). We found the mean value quality of life patients with polyneuropathy to be low in our study.

Axonal polyneuropathies has a negative influence on the quality of life. In a study on 90 patients with chronic axonal neuropathy conducted using the Health Survey Questionnaire (RAND-36), Teunissen et al. (36), identified a low quality of life. In our study, when the quality of life was examined according to the pathogenesis of polyneuropathy, it was demonstrated that subparameters of SF-36 (functional, wellbeing, general and global) and WHOQOL-BREF (physical, psychological, social and environmental) mean quality of life the patients with mixed polyneuropathy was lower than the patients with axonal polyneuropathy. This case can be explained by the presence of mixed polyneuropathy together with axonal and demyelination polyneuropathy.

Many studies indicate that a good glycaemic control positively affects the quality of life (8,37–41). On the contrary, some studies could not find any relationship between the quality of life and the level of HbA1c (42,43). In our study, it was demonstrated that the level of HbA1c did not affect the quality of life of patients. However, it is difficult to comment on this result as deteriorated glycaemic control did not negatively affect the quality of life. As a matter of fact, one of the most important factors in the progression of diabetic neuropathy and nephropathy is the bad glycaemic control (44,45). Therefore, although the level of HbA1c seems to not affect the quality of life in a negative way, because of its role in the development of complications, it can be said it has a direct negative effect on the quality of life.

Our results indicated that, diabetic polyneuropathy as diagnosed on ENMG negatively affects the quality of life of the patients. Polyneuropathy with mixed and sensoriomotor type may especially deteriorate the quality of life to a greater extent. This led to the consideration that the level of polyneuropathy affected the quality of life. Evaluation of ENMG is important for the early diagnosis of neuropathy. Especially the patients who had clinical neuropathy, microalbuminuria, long duration of diabetes and female gender; should be specifically targeted for ENMG evaluation and for taking necessary precautions such as strict glycaemic control and strategies to reduce diabetes-related complications (long term) will be useful for improving the quality of life.
